# Discovery of Western European R1b1a2 Y Chromosome Variants in 1000 Genomes Project Data: An Online Community Approach

**DOI:** 10.1371/journal.pone.0041634

**Published:** 2012-07-24

**Authors:** Richard A. Rocca, Gregory Magoon, David F. Reynolds, Thomas Krahn, Vincent O. Tilroe, Peter M. Op den Velde Boots, Andrew J. Grierson

**Affiliations:** 1 Independent Researcher, Saddle Brook, New Jersey, United States of America; 2 Independent Researcher, Manchester, Connecticut, United States of America; 3 Independent Researcher, Portland, Oregon, United States of America; 4 FamilyTreeDNA Genomics Research Center, Houston, Texas, United States of America; 5 Independent Researcher, Edmonton, Alberta, Canada; 6 Independent Researcher, Amsterdam, North Holland, The Netherlands; 7 Sheffield Institute for Translational Neuroscience, University of Sheffield, Sheffield, United Kingdom; University of Cambridge, United Kingdom

## Abstract

The authors have used an online community approach, and tools that were readily available via the Internet, to discover genealogically and therefore phylogenetically relevant Y-chromosome polymorphisms within core haplogroup R1b1a2-L11/S127 (rs9786076). Presented here is the analysis of 135 unrelated L11 derived samples from the 1000 Genomes Project. We were able to discover new variants and build a much more complex phylogenetic relationship for L11 sub-clades. Many of the variants were further validated using PCR amplification and Sanger sequencing. The identification of these new variants will help further the understanding of population history including patrilineal migrations in Western and Central Europe where R1b1a2 is the most frequent haplogroup. The fine-grained phylogenetic tree we present here will also help to refine historical genetic dating studies. Our findings demonstrate the power of citizen science for analysis of whole genome sequence data.

## Introduction

Population geneticists are in short supply, and most are involved in the important hunt for genetic factors responsible for susceptibility to disease [1]. Naturally, studies that address genetic susceptibility are prioritized over other types of genetic research. As a result, these studies free up little in the way of both human and monetary resources dedicated to the application of genetics to the history of the human species [2], [3]. The study of human phylogenetics is where the practice of citizen science can be a valuable resource to the scientific community. In the name of citizen science, the authors of this study pooled together their resources and volunteered their time to data mine the full genomes of over a thousand samples that were made freely available through the 1000 Genomes Project [4].

The 1000 Genomes Project is the first project to sequence the genomes of a large number of people. The plan for the full project is to sequence about 2,500 samples; however, only the data sets of 1,197 samples from 13 populations were available in early 2011. It is estimated that over 100 million European men belong to haplogroup R1b1a2 (M269) [5], and that greater than 70% of western European men belong to the specific clade defined by SNP L11 [6]. The focus of the work was finding phyologenetic Y-DNA variants in the two largest sub-clades of L11: S116/P312 (rs34276300) and U106/S21 (rs16981293). To illustrate the need for more refined testing, no study to date has tested for L48 (rs13303755), the most frequent variant in the U106 branch of L11 (http://www.familytreedna.com/public/u106/), and published studies still show ambiguous S116 (xU152, L21) as the most frequent variant in Spain, Portugal and parts of France [6], [7].

Recently, there has been much controversy in the dating and dating techniques used to identify the geographical distribution of R1b1a2 by way of microsatellite variance or diversity [7]. The paucity of haplogroup defining genetic markers has meant that these microsatellite-derived dating calculations have to be conducted without regard to lower level phylogenetic relationships, and therefore erroneously compare populations that may be phylogenetically distant. By identifying the lower level branches of the R1b1a2 phylogenetic tree, more accurate dating of truly related haplogroups will be possible.

## Results

**Figure 1:**
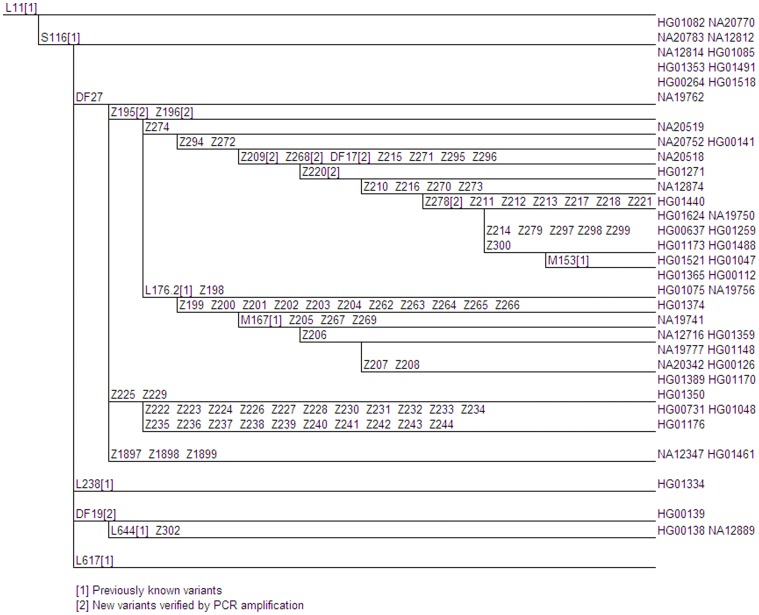
Proposed S116 (xU152, L21) Phylogenetic Tree. Genetic variants are indicated on branches, and branch lengths are not proportional to the number of mutations or the age of the variant. Phylogenetically equivalent markers are shown in alphabetical and numerical order. Full details of these variants are shown in Table S1. The positions of 1000 Genomes samples are given at the tips of the branches.

**Figure 2:**
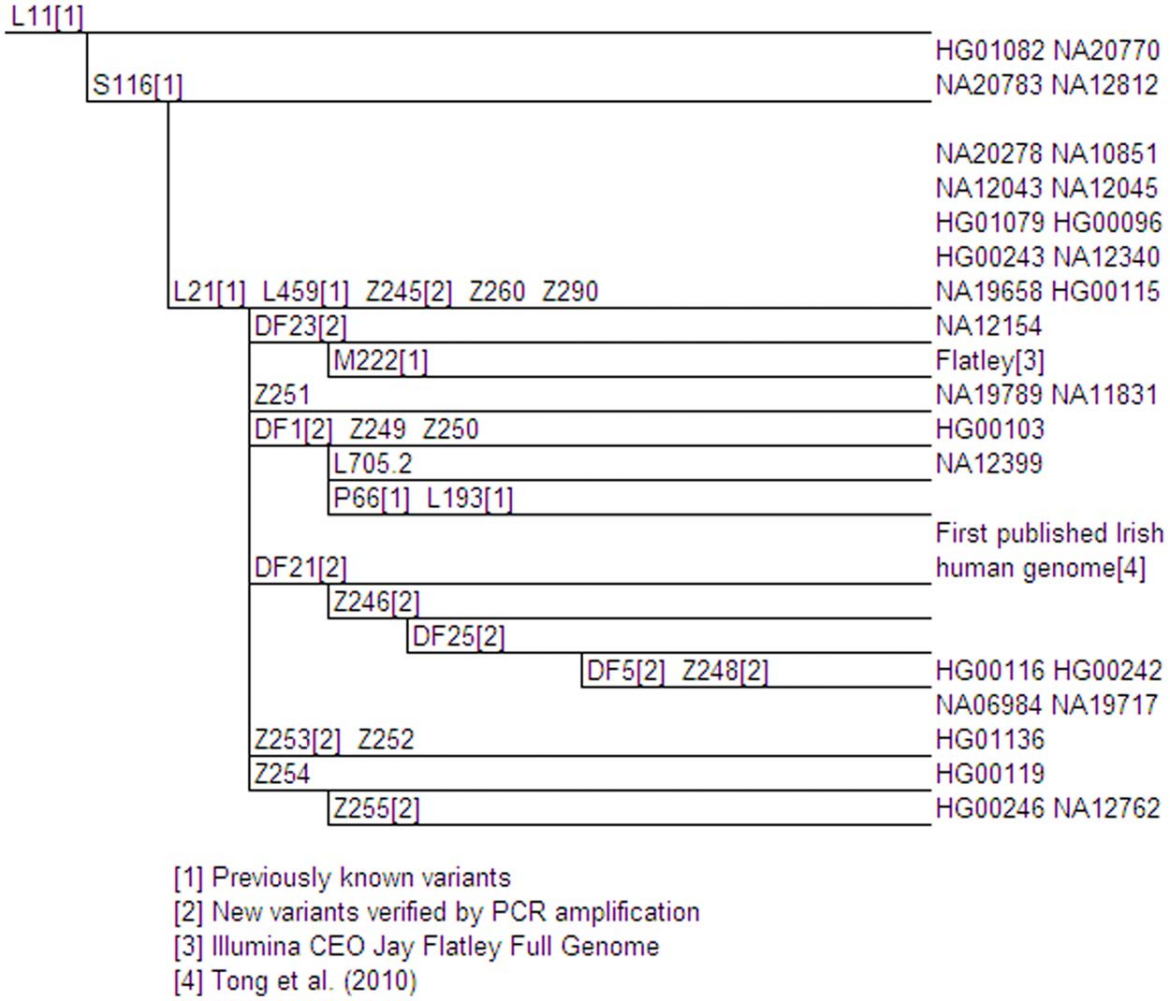
Proposed L21 Phylogenetic Tree. Genetic variants are indicated on branches, and branch lengths are not proportional to the number of mutations or the age of the variant. Phylogenetically equivalent markers are shown in alphabetical and numerical order. Full details of these variants are shown in Table S1. The positions of 1000 Genomes samples are given at the tips of the branches.

**Figure 3:**
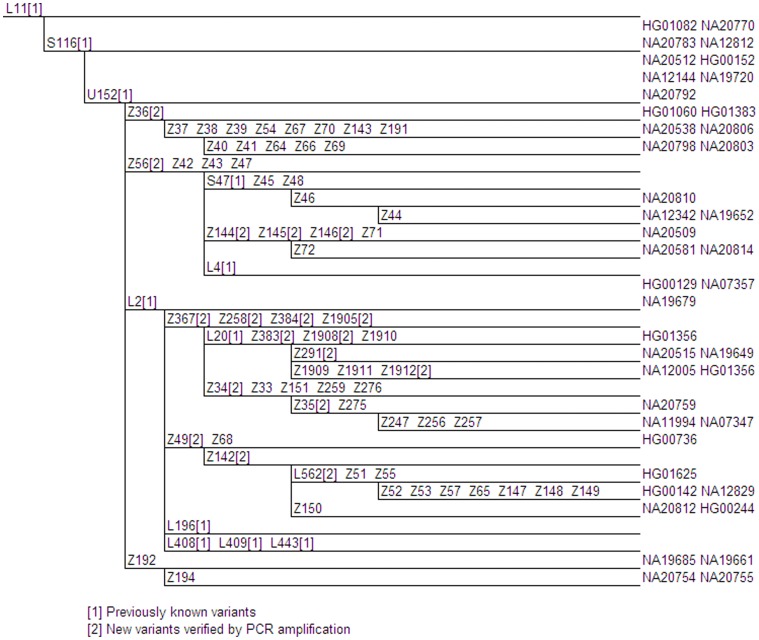
Proposed U152 Phylogenetic Tree. Genetic variants are indicated on branches, and branch lengths are not proportional to the number of mutations or the age of the variant. Phylogenetically equivalent markers are shown in alphabetical and numerical order. Full details of these variants are shown in Table S1. The positions of 1000 Genomes samples are given at the tips of the branches.

**Figure 4:**
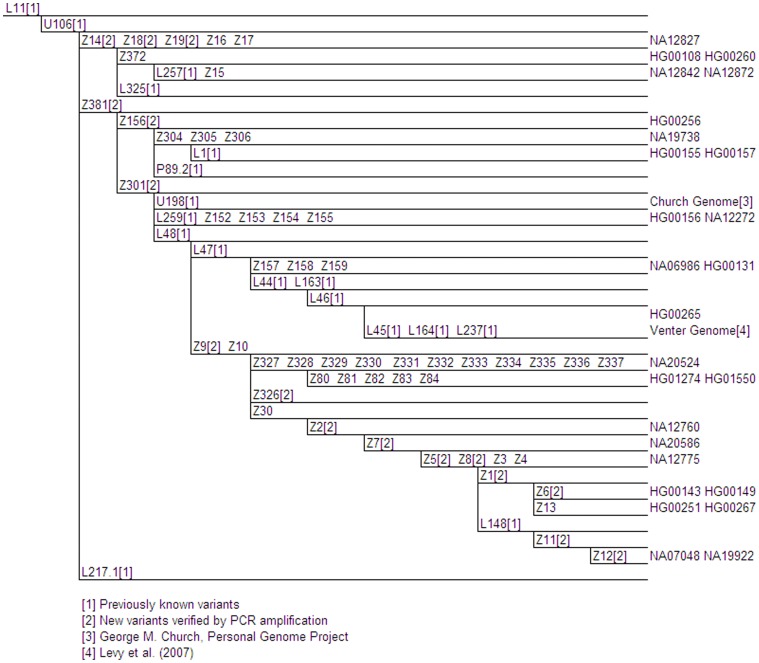
Proposed U106 Phylogenetic Tree. Genetic variants are indicated on branches, and branch lengths are not proportional to the number of mutations or the age of the variant. Phylogenetically equivalent markers are shown in alphabetical and numerical order. Full details of these variants are shown in Table S1. The positions of 1000 Genomes samples are given at the tips of the branches.

By analysing the 1000 Genomes Project Phase 1 data set of 1,197 individuals, we identified 135 samples bearing the L11 SNP. Excluding Finland, which has a low L11 frequency, approximately 50% of the remaining datasets comprising European populations CEU (CEPH Utah residents with Northern and Western European ancestry), GBR (British in England and Scotland), IBS (Iberian populations in Spain) and TSI (Tuscans in Italy) were derived at L11 [8]. An additional source of L11 derived datasets came from Latin American populations MXL (Mexican Ancestry in Los Angeles, California), PUR (Puerto Rican in Puerto Rico) and CML (Colombian in Medellin, Colombia) and to a minor extent, the ASW (African Ancestry in Southwest US) population. L11 is divided into two major sub-clades: S116 and U106. A large majority of L11 samples belong to subclade S116 (109 out of 135 or 81%). Using SAMtools and filtering methodology described in the methods section, we identified more than 200 putative non-singleton novel genetic variants in the 135 R1b1a2-L11 samples. The resulting R1b1a2-L11 phylogenetic tree based on Phase 1 1000 Genomes data is presented in Figures 1, 2, 3 and 4.

### R1b1a2-S116 Sub-Haplogroups

Forty-two of the 49 samples that would previously have been categorized as belonging to unspecified S116(xU152,L21) were derived for the DF27 SNP (Figure 1). The majority of the DF27 derived samples (71%) were from Latin American/Iberian populations (CLM, IBS, MXL, PUR). This variant is therefore likely to account for the majority of previously unclassified S116(xU152,L21) reported in Iberia and some areas of France [7]. Below DF27, we identified 17 subclades and a total of 73 novel mutations. We were also able to place previously known SNP Z278 (rs1469371) into a phylogenetic context and show for the first time a close relationship between markers M153, M167/SRY2627 and L176.2. In the branch of S116 defined by DF27 a total of six new SNPs were validated by PCR amplification and Sanger sequencing. A description of the locations and sequences of all primers developed for validation experiments is given in Table S1.

A smaller subclade of S116(xU152,L21,DF27) consisting of two GBR samples and a single CEU sample was defined by DF19. Another S116(xU152,L21,DF27) GBR sample carried the previously unpublished L238 SNP. The northern European origin of the sample is not surprising given that all previously known L238 samples have been from Scandinavia (http://www.familytreedna.com/public/atlantic-r1b1c/).

Marker L21/M529/S127 (rs11799226) is currently perhaps the most clearly geographically localized of the major L11 sub-haplogroups, with a high frequency in the British Isles and Brittany [7]. Twenty-three samples were found to be derived at marker L21 (Figure 2). Unsurprisingly, most L21 derived samples were either from GBR (34.8%) or from the CEU (30.4%) population, which itself is most similar to samples from the Netherlands and the UK [9]. The remainder of the samples were from MXL, CLM, PUR and ASW. We were able to group 13 new variants into 13 subclades of L21. Ten of the new markers have been confirmed by PCR amplification and Sanger sequencing. Some of these were found by comparing L21 derived 1000 Genomes Project samples with two publicly available genomes (see Materials and Methods). The previously known marker M222 was re-positioned within the L21 group below DF23, and new sub-haplogroups were defined by Z251, DF1, DF21 (rs138322855), Z253 and Z255. DF21 and DF1 lie upstream of a number of new and previously identified variants, respectively (Figure 2).

The S116 downstream marker U152/S28 (rs1236440) defines the most common Y chromosome haplogroup in northern and central Italy, Switzerland, eastern France and Corsica [7]. The genomes of 36 U152 derived males were analyzed (Figure 3). Samples from the Tuscany region of Italy were found to have a high frequency of U152 (29.4%). This correlates well with the frequency of 32.4% previously reported in central Italy and the 32.1% found in Corsica [7], [10]. We were able to further refine U152 into 26 subclades. Fifty-four new variants were found in all, 11 of which have been validated by PCR amplification and Sanger sequencing. Additionally, SNPs Z367 (rs7067387), Z258 (rs9785865), Z384 (rs28819996), Z1905 (rs7892878), Z383 (rs34173183), Z1908 (rs11799240) and Z1912 (rs4893798) were placed below U152 in the phylogenetic tree for the first time. To date all U152 samples with DYS492 = 14 appear to be in the Z56 subbhaplogroup, including, but not limited to those derived at marker L4 (Family Tree DNA R1b-U152 Project: http://www.familytreedna.com/public/R1b-U152/default.aspx).

Of the four remaining S116(xDF19, DF27, L21, L238, L617, U152) samples, three were missing data for DF27 and a fourth had a weak ancestral read.

### R1b1a2-U106 Sub-Haplogroups

Sub-haplogroup U106/S21/M405 (rs16981293) (Figure 4) makes up the other half of the L11 story in Europe. It is the most common R1b1a2 marker in central Europe, and is by far the most frequent SNP in the Netherlands and Belgium [7], [11]. Twenty-six samples were found derived at marker U106. As was the case with marker L21, GBR (42.3%) and CEU (42.3%) population samples made up a large percentage of U106 samples. The U106 tree now consists of 30 downstream subclades. We found 49 novel SNPs in this group and were able to place known SNPs Z301 (rs35121273) and Z381 (rs34001725) into the U106 sub-haplogroup. Fifteen of these new variants were confirmed by PCR amplification and Sanger sequencing. The known markers L48 (rs13303755) and U198/S29 (rs17222279) were also relocated to lower branches in the U106 tree. George Church’s genome allowed us to place U198 with respect to other 1000 Genome Project samples (http://www.personalgenomes.org/pgp10.html).

### The Validated L11 Phylogeny

**Figure 5:**
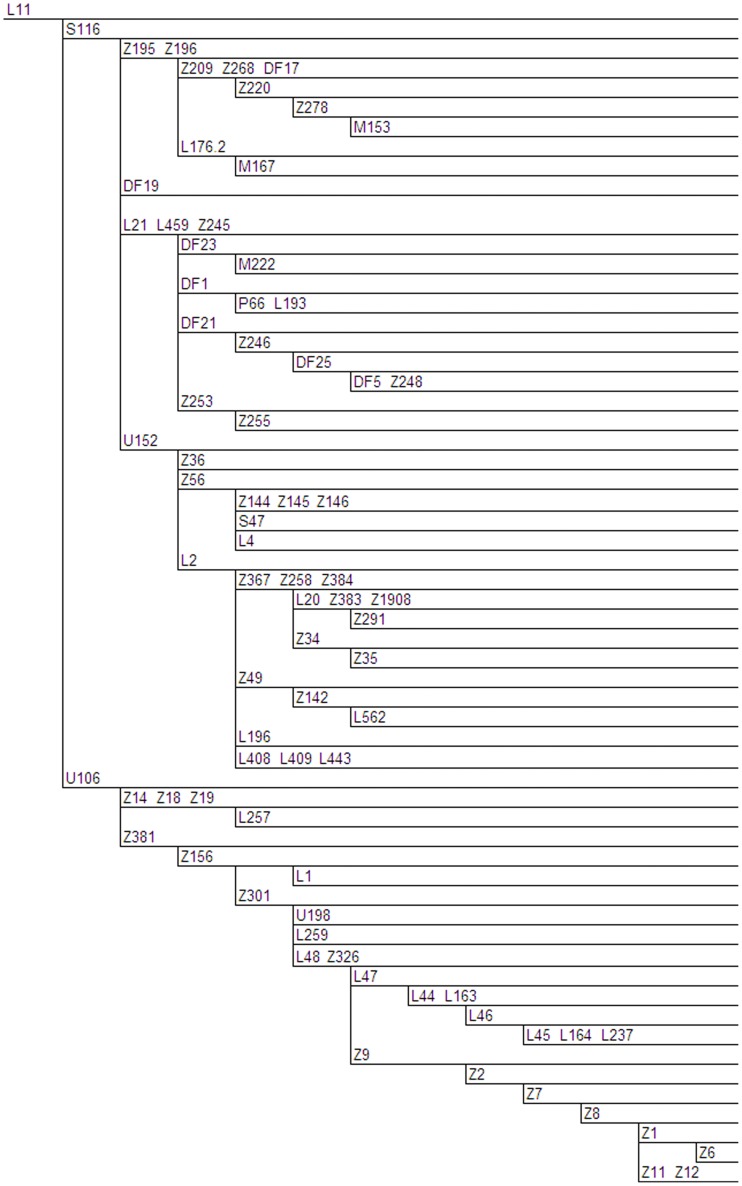
Validated L11 Phylogeny. Genetic variants that have been validated by Sanger sequencing are indicated on branches, and branch lengths are not proportional to the number of mutations or the age of the variant. Phylogenetically equivalent markers are shown in alphabetical and numerical order. Full details of these variants are shown in Table S1.

Despite using a number of filtering steps to reduce false discovery rates, it remains formally possible that a number of variants may be artefacts, or are refractory to Sanger sequencing. Thus we also present here a summary of the L11 phylogeny containing all polymorphisms that have been independently validated to date by PCR amplification and Sanger Sequencing (Figure 5).

## Discussion

The Y chromosome haplogroup tree has evolved from a collection of 243 unique polymorphisms in 2002, to several thousand markers today [12] (http://www.isogg.org/tree/). The availability of high quality whole-chromosome sequence data from thousands of individuals has expedited the discovery of new polymorphisms, and the open access nature of the 1000 Genomes Project has enabled public volunteers to practice citizen science to trail-blaze a new haplogroup tree for R1b1a2-L11.

The R1b1a2-L11 haplogroup is prevalent in Western Europe, and this has led to conflicting opinions about the spread of populations from east to west since the Neolithic [5], [6]. Busby et al. stated that coalescence estimates explicitly depend on the STRs that one uses [7], thus the use of relatively small numbers of microsatellite markers in previous studies may have been problematic, but a more precise sub-classification of R1b1a2-L11 by SNPs will help to resolve these controversies. Better SNP resolution could also answer questions regarding the use of evolutionarily effective and germ line mutation rates [13]. Given the regional affinities of L21, U152, U106 and now DF27, we can already see where studying their downstream variants can help answer questions whose answers have long eluded archaeologists and linguists alike, especially if some of the newly defined haplogroups prove to be geographically clustered.

In Iberia, previous data suggests evidence of gene flow between Basques and Catalan speaking populations based on the distribution of M167 (SRY2627) [14], [15], [16]. Our placement of M153, a marker that has been linked to Basque populations [17], and SRY2627 as downstream variants of DF27 further illustrates this close relationship. The position of M153 many levels down on the phylogenetic tree also shows its relative youth within R1b1a2, as does its low STR diversity [18] (Family Tree DNA M153 Project: http://www.familytreedna.com/public/R-M153_The_Basque_Marker/default.aspx).

The concentration of Latin American/Iberian samples derived at DF27 shows the geographical importance of this marker in Iberia. That only Latin American samples were members of the DF27 subclade defined by the mutations Z225 and Z229 further illustrates strong Iberian ties and is consistent with the possibility of colonial era founders in the Americas [19].

Genetic approaches offer unique possibilities to resolve longstanding historical and archaeological dilemmas. Analysis of subclades defined by the new genetic markers reported here could help resolve some of these. As marker L21 has its highest frequencies in areas where insular Celtic languages once dominated, and some areas where they are still spoken today, there is no doubt that understanding it subclades will be instrumental in any debate regarding Celtic origins [20]. Given that U152 is the most frequent marker in northern and central Italy, its new subclades should prove valuable in resolving the fundamental problems of establishing the origin of IE groups in Italy [20], [21], [22]. Marker U106 and its subclades, so prominent along the Rhine River, can give insights into the spread of Germanic languages. And finally, any future testing of ancient Y-DNA Bell Beaker skeletons should start testing for these markers as Bell Beaker skeletons have already turned up derived at marker R1b-M269 with no downstream markers tested [23]. The discovery of these new markers is only the first step. While commercial testing of some of these markers has commenced, the real benefit will come from typing a large number of L11 derived individuals of phylogeographic background.

We are currently witnessing the resolution of two of the biggest impediments to human population genetics. The first is that of cost, with the $1,000 full genome sequence seemingly around the corner [24]. The second is the extraction of ancient Y-DNA, which is already becoming a reality [25], [26]. The more economically feasible it is to sequence the entire genome of existing humans and the easier it is to apply the novel variants found in this study and compare them to ancient Y-DNA, the quicker our historical, archaeological and linguistic questions will be answered in regard to the populating of Western Europe by R1b1a2.

The progress we have made towards resolving the L11 phylogeny has been significant considering that none of the authors of this manuscript have ever met, nor spoken on the phone. Open source data, open source tools, and open forums enable research collaborations to blossom. In fact, other citizen scientists are currently using similar approaches to find groundbreaking SNPs in other branches of the Y chromosome haplogroup tree.

## Materials and Methods

The main mode of communication was through the DNA-Forums website (DNA-Forums, A Genetic Genealogy Community, http://dna-forums.org). It was here that general information on data mining techniques and the discovery of new variants was shared and discussed amongst team members. The ISOGG phylogenetic tree was used as a starting point for phylogenetic placement as it was found to be the most current (http://www.isogg.org/tree/ISOGG_HapgrpR.html).

SAMtools [27], and related utilities were used to query low coverage (2–4x) datasets from publicly accessible FTP sites at the European Bioinformatics Institute (ftp://ftp.1000genomes.ebi.ac.uk/vol1/ftp/) and the National Center for Biotechnology Information (ftp://ftp-trace.ncbi.nih.gov/1000genomes/ftp/). Using SAMtools tview, samples were screened for the L11 mutation. BAM index files were then called for each L11 derived sample along with a control group of non-R1b1a2 samples using SAMtools mpileup. The resulting BCF file was then converted into VCF format by using the bcftools vcfutils.pl script. The resulting VCF file was opened in MS Excel for visual identification of potential SNPs downstream of L11.

Novel variants were filtered by verifying that all non-L11 control samples bore the ancestral allele, and by identifying at least two L11 samples that carried the same derived allele. While hundreds of singletons were found, they were not catalogued. Variants which had heterozygous allele calls were disregarded, as were those with phylogenetic inconsistencies that more than likely arose in duplicated or recombining regions of the Y-chromosome. Once a variant fitting these filtering criteria was found, it was further verified by making individual calls to the respective chromosome Y position using the SAMtools tview. These positions were also cross-referenced against the Family Tree DNA Y Chromosome Browser (http://ymap.ftdna.com) to determine whether the variants were novel.

A master list of new variants was kept in a centralized spreadsheet stored in Google Docs (https://docs.google.com), a freely available cloud storage service. This made uploading, storing and organising the data easier to manage and readily available to any citizen scientist with access to the Internet.

Primers (see Table S1) were designed for a number of variants of genealogical or phylogenetic importance, using Primer3 [28], or the National Center for Biotechnology Information online Primer-BLAST tool (http://www.ncbi.nlm.nih.gov/tools/primer-blast/). Variants were validated using PCR and Sanger sequencing.

Several other publicly available genome data sets were used for the identification of new variants. These included Jay Flatley’s genome (http://aws.amazon.com/datasets/3357), the first Irish genome [29] and the genomes of Henry Louis Gates Jnr and Snr (http://snp.med.harvard.edu).

## Supporting Information

Table S1 **Details of all genetic variants identified using 1000 Genomes data details of PCR primers used to validate variants using Sanger Sequencing.**
(XLSX)
