# Correction: Discovery of Western European R1b1a2 Y Chromosome Variants in 1000 Genomes Project Data: An Online Community Approach

**DOI:** 10.1371/annotation/5d0f79cd-30a5-45b7-aaff-4e27892a9d20

**Published:** 2012-08-15

**Authors:** Richard A. Rocca, Gregory Magoon, David F. Reynolds, Thomas Krahn, Vincent O. Tilroe, Peter M. Op den Velde Boots, Andrew J. Grierson

In the R1b1a2-S116 Sub-Haplogroups section of Results, the correct alias for L21/M529 is S145, not S127.

In Figure 5, the positioning of Z253 vs Z255 was confirmed by PCR amplification and Sanger sequencing; the two SNPs are mutually exclusive and are located at a peer level under R-L21. This is also consistent with the placement of the two SNPs as shown in Figure 2 of this article. Also, the positioning of Z301 vs Z156 was confirmed by PCR amplification and Sanger sequencing; the two SNPs are mutually exclusive and are located at a peer level under R-Z381. This is also consistent with the placement of the two SNPs as shown in Figure 4 of this article.

Figure 4 should show Z11 directly below Z5/Z8 and Z12 below Z11 and L148 below Z12.

In Figure 3, HG01356 is listed as both L20 and Z1909. It should only appear on the Z1909 line.

